# Differential Retention and Loss of a Mycotoxin in Fungal Evolution

**DOI:** 10.3390/toxins17060311

**Published:** 2025-06-19

**Authors:** Lin Chen, Ziying Yan, Bolei Yang, Bowen Tai, Weizhao Li, Erfeng Li, Gang Wang, Fuguo Xing

**Affiliations:** 1 Comprehensive Utilization of Edible and Medicinal Plant Resources Engineering Technology Research Center, Zhengzhou Key Laboratory of Synthetic Biology of Natural Products, Huanghe Science and Technology College, Zhengzhou 450006, China; lchenchina@163.com; 2 Key Laboratory of Agro-Products Quality and Safety Control in Storage and Transport Process, Ministry of Agriculture and Rural Affairs, Institute of Food Science and Technology, Chinese Academy of Agricultural Sciences, Beijing 100193, China; yanziyingyy@163.com (Z.Y.); yangbolei@caas.cn (B.Y.); taibowen@caas.cn (B.T.); liweizhao@caas.cn (W.L.); xingfuguo@caas.cn (F.X.); 3 Horticulture and Landscape College, Tianjin Agricultural University, Tianjin 300392, China

**Keywords:** ochratoxin, gene cluster, evolution, degeneration

## Abstract

Ochratoxin A (OTA) is designated as a mycotoxin and is regulated worldwide due to its harmful effects on humans and animals, but the evolutionary history and ecological significance of OTA in fungi remain poorly understood. Phylogenetic analysis suggested that *Aspergillus* and *Metarhizium* obtained an ancient OT cluster, which evolved independently, followed by horizontal OT transfer from *Aspergillus* to *Penicillium*. The varying presence of functional, absent and pseudogenized OT genes across *Aspergillus* species revealed that this cluster is undergoing a degeneration process in this genus. Furthermore, the cyclase OtaY in the OTA cluster is likely derived from bacteria, which was revealed by phylogenetic analysis. This is the first attempt to investigate the ecological significance of OTA in fungi, suggesting that it may be nonfunctional in *Aspergillus* spp. and has undergone multiple forms of loss during evolution.

## 1. Introduction

The utility of fungi in industrial, nutritional and medical endeavors, as well their production of secondary metabolites (SMs) toxic to humans, animals and plants, has huge consequences on human society. Ochratoxins (OTs) are a group of mycotoxins contaminating a variety of food and feed [1]. OTA is subject to regulatory limits in several countries and regions, such as the European Union [2] and China [3], due to its nephrotoxicity, hepatotoxicity, teratogenicity, immunotoxicity and the potential carcinogenicity (group 2B) [4].

**Figure 1:**
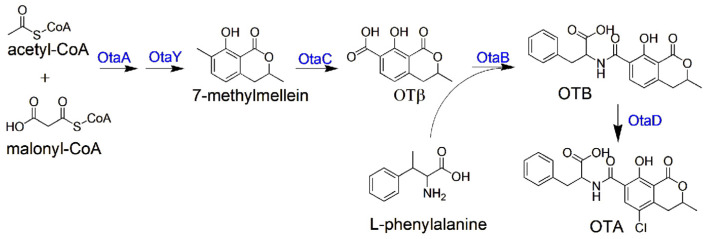
The biosynthetic pathway of OTA.

Fungal genomes characteristically package the complete set of genes needed to synthesize SMs into tightly linked chromosomal segments known as biosynthetic gene clusters (BGCs) [5]. Orthologous gene clusters identified through a pan-genomic analysis of OTA producers suggest the existence of core enzymatic machinery essential for this secondary metabolite’s production [6]. Briefly, the OTA assembly line initiates with OtaA (PKS), orchestrating polyketide chain elongation from CoA-activated precursors to yield 7-methylmellein. Oxidative remodeling by OtaC’s P450 system generates the OTβ scaffold, which is subsequently aminoacylated with L-Phe through OtaB’s NRPS machinery. Final maturation via OtaD-mediated halogenation completes the toxic OTA, with OtaR1 governing this enzymatic symphony through transcriptional regulation (Figure 1). Emerging genomic evidence has identified an enzymatic cyclase (*otaY*) flanked by *otaA* and *otaB* in the biosynthetic cluster, which is hypothesized to mediate structural cyclization of the linear polyketide intermediate during OTA assembly [7,8].

Mycotoxins are designated according to their harmful effects on humans and animals, while their ecological significance to the producing fungi and their evolutionary processes that have shaped mycotoxin diversity are largely unknown. Accruing data, however, suggest that mycotoxins and other fungal SMs are fitness factors that help fungi withstand abiotic or biotic stress [9,10]. Although the effects of OTA on humans and animals are clear, its evolutionary history and ecological significance in fungi remain vague.

Given the vital roles of fungal SMs, the evolutionary history by which the SMs were created has garnered much interest. Horizontal gene transfer (HGT) is a widely recognized mechanism involved in the formation and maintenance of SM BGCs [11]. The HGT hypothesis is consistent with the “selfish cluster/operons” model of gene cluster evolution [12,13]. The identification of HGT relies on multiple evidentiary criteria, including but not limited to discordant phylogenetic placement patterns, an unexpectedly high sequence homology with organisms outside the expected evolutionary clade and statistically supported conflicts between gene tree topologies and species trees [14]. Recent analyses in fungi genome evolution have provided increasing evidence of HGT of SMs between fungi, such as the ACE1 [15] and DEP [16] BGCs among *Sordariomycetes*, *Eurotiomycetes* and *Dothideomycetes* and the aflatoxin-like cluster between *Aspergillus* and *Podospora* [17]. Comparative genomics analyses demonstrate maintained syntenic conservation of the OTA BGC across *Penicillium* and *Aspergillus*, despite divergent chromosomal positioning evidenced by heterogeneous adjacent genomic contexts [18]. These findings provide compelling evolutionary evidence for the cluster’s orthologous origin while suggesting its probable dissemination through HGT events. However, research into the origin and evolution of this gene cluster in OTA-producing fungi remains severely limited.

Currently, fungal species belonging to *Aspergillus* and *Penicillium* are known to produce OTA [1]. We also found that three species of the distant phylogenetic taxon *Metarhizium* contained an expressed OT BGC lacking an *otaD* gene but did not produce the expected OTB [19]. The intricate regulatory networks governing fungal SM biosynthesis present significant mechanistic complexities. Notably, despite the undetectable production of OTB metabolites, the persistence of an intact OTB BGC in *Metarhizium* spp. remains unexplained. Investigating the evolutionary persistence of OTA biosynthesis across fungal taxa may provide critical insights into the ecological drivers maintaining such metabolic traits. In this study, a phylogenetic analysis indicated that *Aspergillus* and *Metarhizium* acquired a prehistoric OTA cluster through separate evolutionary processes, and subsequently, horizontal OT transfer occurred from *Aspergillus* to *Penicillium*. The diverse occurrence of functional, nonexistent and pseudogenized OTA genes among *Aspergillus* species indicates that this cluster is undergoing a degeneration process within this genus.

## 2. Results

### 2.1. HGT Contributes to the Acquisition of OT Genes

**Figure 2:**
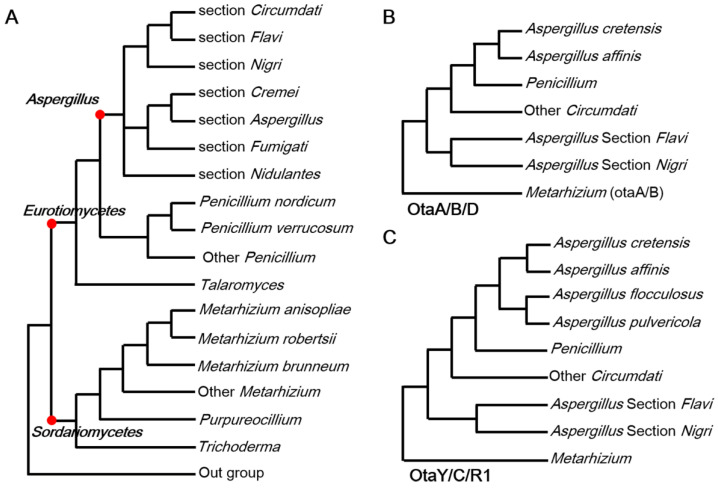
Maximum likelihood phylogenies for species and OTA homologs. (**A**) Fungal species phylogeny. (**B**) The phylogeny pattern of OtaA/B/D. *Metarhizium* spp. do not contain OtaD. (**C**) The phylogeny pattern of OtaY/C/R1.

The discontinuous distribution of the OT BGC in the Eurotiomycetes and its presence in some Metarhizium species raised the possibility that the OT BGC originated via HGT in some species (Figure 2A and Figure S1). Gene-by-gene phylogenetic analyses were performed to decipher the evolutionary route of OTA using homologs identified across fungi (Figure S2). The HGT hypothesis predicts incongruences between taxonomic phylogenies of the species versus the BGCs [20]. A phylogenetic analysis revealed two distinct topological patterns. OtaA, B and D support an adjacent group between the *Penicillium* clade and the *Aspergillus cretensis* clade (*A. cretensis* and *A. affinis*) (Figure 2B). OtaY, C and R1 also support an adjacent group between the *Penicillium* clade and the slightly larger *A. cretensis* clade comprising *A. cretensis*, *A. affinis*, *A. flocculosus* and *A. pulvericola* (Figure 2C). Overall, the phylogenetic placement of *Penicillium* based on OT genes is in conflict with its expected position based on species phylogeny and, further, breaks up the *Aspergillus* sequence clan. The six gene phylogenies suggest the Penicillium OT BGC arises from an *A. cretensis* ancestor after its divergence from section *Circumdati*.

In addition to *Aspergillus* and *Penicillium* species, three species of the distantly related taxon *Metarhizium* have an expressed OT-like BGC but lack an *otaD* gene [19]. The placement, which was based on five OT proteins (OtaA, Y, B, C and R1), was consistent with *Metarhizium* species phylogeny, thus indicating that OT BGCs in *Eurotiomycetes* and *Sordariomycetes* have undergone a long history of independent evolution. We would predict that if *Metarhizium* was the recipient of HGT from *Aspergillus*, then the OT genes would show an expected close relationship to the donor lineage, or vice versa. Therefore, we suggest that HGT events have not occurred between *Metarhizium* and *Aspergillus* (or *Penicillium*). Another hypothesis is that some ancestor of *Metarhizium* and *Aspergillus* contained an OT BGC, and the evolution of the cluster was accompanied by the divergence of the species. This “vertical descent” hypothesis is unlikely because it requires massive gene losses in species across *Eurotiomycetes* (including 1332 species with genomic sequences) and *Sordariomycetes* (including 3003 species with genomic sequences). In conclusion, our results support the view that OT BGCs evolved independently in *Aspergillus* and *Metarhizium*, although we cannot clearly discern the respective origin(s) of this BGC for either genus.

**Figure 3:**
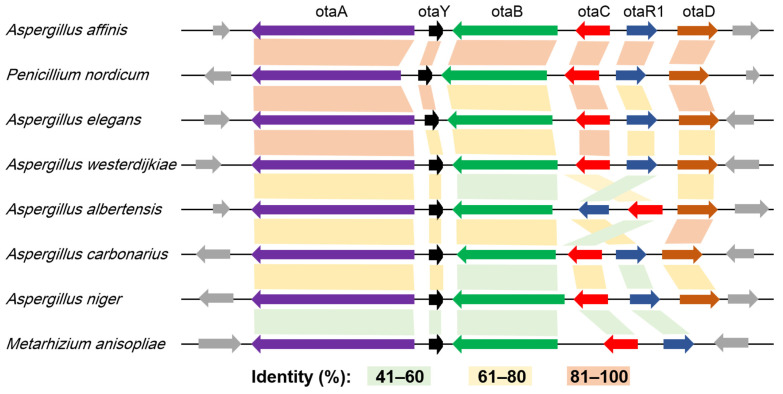
Alignment comparison analysis derived from different fungal species. Gray arrows denote genes flanking the OTA BGCs.

An alignment comparison analysis was conducted on OTA BGCs derived from eight fungal strains representing diverse taxonomic groups: *Aspergillus* section *Circumdati* (*Aspergillus affinis*, *A. elegans* and *A. westerdijkiae*), *Aspergillus* section *Flavi* (*A. albertensis*), *Aspergillus* section *Nigri* (*A. carbonarius* and *A. niger*), *Penicillium nordicum* and *Metarhizium anisopliae*. As illustrated in Figure 3, homologous proteins exhibited sequence identity exceeding 40%, while flanking genes adjacent to OTA BGCs demonstrated functional divergence and lacked homology across different species. Notably, higher sequence identity was observed between the OTA biosynthetic proteins of *P. nordicum* and those from *Aspergillus* section *Circumdati* (mean 85%) compared to the identity between *Aspergillus* sections *Circumdati* and *Flavi* (mean 63%). These comparative genomic patterns suggest potential HGT of OTA BGCs among phylogenetically distant fungal taxa.

### 2.2. Degeneration of OT Cluster in Aspergillus

**Figure 4:**
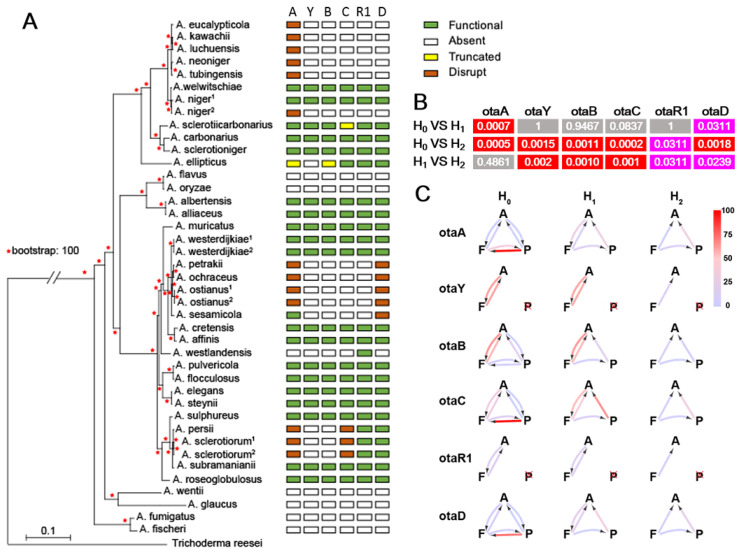
Degeneration of OTA cluster in *Aspergillus*. (**A**) Maximum likelihood relationship inferred using 2130 orthologs from 42 species and variation of genes in OTA clusters shown in character grid. (**B**) Likelihood ratio tests (LRTs) of the transition models (*p*-value). The statistical difference (*p* < 0.05) and significant difference (*p* < 0.01) are represented by pink and red color, respectively. (**C**) Transition rate of genes among functional (F), absent (A) and pseudogenized (P) states. The truncated and disrupted genes are included in the pseudogenized state. Arrow color represents the estimated transition rate.

**Figure 5:**
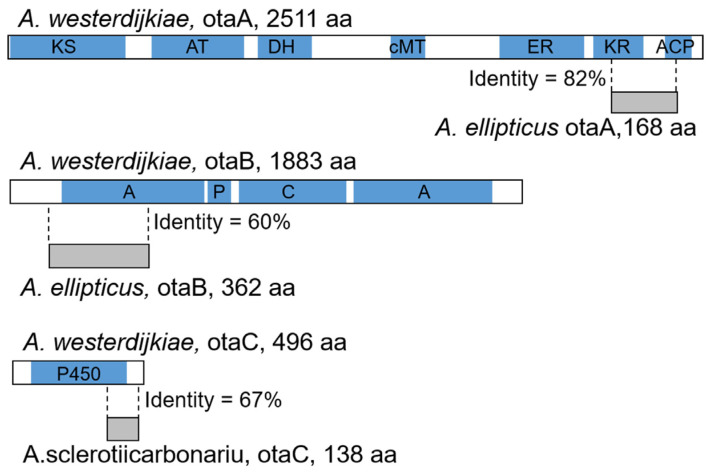
Comparison of truncated and functional protein sequences; blue blocks represent the protein domains.

In the course of searching for OT BGCs, some species demonstrated truncated OT genes, similar to findings of BGC degeneration in other fungi, such as in the xanthocillin and citrinin BGCs in various *Aspergillus* and *Penicillium* spp. [21], the bikaverin BGC in *Botrytis* spp., which was horizontally transferred from *Fusarium* spp. [22], and the citrinin and monacolin K BGCs in *Monascus* spp. [23]. Focusing on *Aspergillus* spp. (Table S1) and shown in Figure 4A, the degeneration of the OT BGC fell into several patterns. Species in the *Aspergillus eucalypticola* clade retain only a small part of OtaA. Other truncations are found in OtaC in *A. sclerotiicarbonarius* (138 amino acids), and in both OtaA and OtaB (168 amino acids and 362 amino acids, respectively) in *A. ellipticus*. As shown in Figure 5, a functional *otaA* with biological activity consists of multiple domains including Ketosynthase (KS), Acyltransferase (AT), Dehydratase (DH), C-Methyltransferase (cMT), Enoylreductase (ER), Ketoreductase (KR) and Acyl Carrier Protein (ACP). However, *otaA* in *A. ellipticus* retains only partial sequences of the KR and ACP domains, exhibiting 82% sequence identity with its homolog in *A. westerdijkiae*. Similarly, *otaB* from *A. ellipticus* and *otaC* from *A. sclerotiicarbonarius* demonstrate relatively high sequence identity (60% and 67%, respectively) with their corresponding homologous proteins in *A. westerdijkiae*. However, the three putative truncated proteins were considered nonfunctional given the loss of functional domains. In *Aspergillus petrakii*, *A. ochraceus* and *A. ostianus*, the BGC was nearly entirely lost, with only a small part of OtaA and OtaD remaining. In *A. sesamicola*, OtaA was retained, while other genes except part of OtaD were deleted. *A. westlandensis* retained an orphan *otaR1* gene. *A. persii*, *A. sclerotiorum* and *A. sclerotiorum* retained *otaR1* and *otaD*, and a long DNA fragment consisting of complete sequences of *otaY* and *otaB* but only parts of *otaA* and *otaC* was lost. Overall, the degeneration of the OT BGC is primarily found in sections *Circumdati* and *Nigri*.

**Figure 6:**
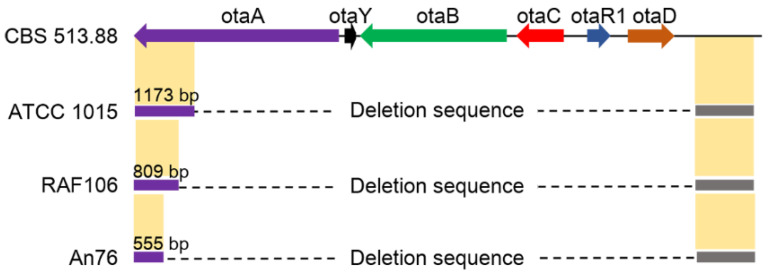
The patterns of OTA loci in *A. niger* strains.

In addition, a total of 15 *Aspergillus niger* genomes from NCBI were analyzed for the intraspecific differentiation of OT clusters. As shown in Figure 6, the model *A. niger* strain CBS 513.88 contains an intact OT BGC, while strain ATCC 1015 lost a large gene segment about 21 Kb from *otaA* to *otaD*. Among the 15 strains, H915-1, ATCC13496, A1, L2, LDM3 and SH2 demonstrated a pattern similar to that of CBS 513.88, and ATCC 10864, FDAARGOS_311, FGSC_A1279, JSC093350089, MOD1_FUNGI2, NRRL3 and N402 demonstrated a pattern similar to that of ATCC 1015. The cleavage sites in the *otaA* gene of *A. niger* strains RAF106 and An76 differ from those in the reference strain ATCC 1015, suggesting the presence of polymorphisms in gene deletions within the OTA BGC across distinct *A. niger* strains. These results indicate that this BGC is experiencing a decay process in *A. niger*.

To model the gain and loss of OT genes across *Aspergillus* species, we calculated the gene transition rate among functional (F), absent (A) and pseudogenized (P, truncated and disrupt) genes under three models: H_0_ (all gene state transitions are permitted), H_1_ (all loss types are permitted, while functional genes can only be acquired from the absent state, representing an HGT) and H_2_ (only gene losses are permitted). The analysis showed different transition rate patterns for each OT gene. In gene-by-gene likelihood ratio tests (LRTs) (Table S2), H_2_ showed significant differences with H_0_ and H_1_, while there was no statistic difference between H_0_ and H_1_ (Figure 4B). This suggested a gene decay model (H_2_) approximated the evolution of this cluster in *Aspergillus*. From the perspective of gene decay (Figure 4C and Table S2), *otaA* had the lowest rate of gene deletion (F to A), but the highest rate of pseudogene loss (F to P). That was consistent with the multiple disruptions seen in *otaA*. These results indicate that there was no HGT among *Aspergillus* species, and the OT cluster is undergoing a degeneration process.

### 2.3. Fungi May Acquire otaY from Bacteria

**Figure 7:**
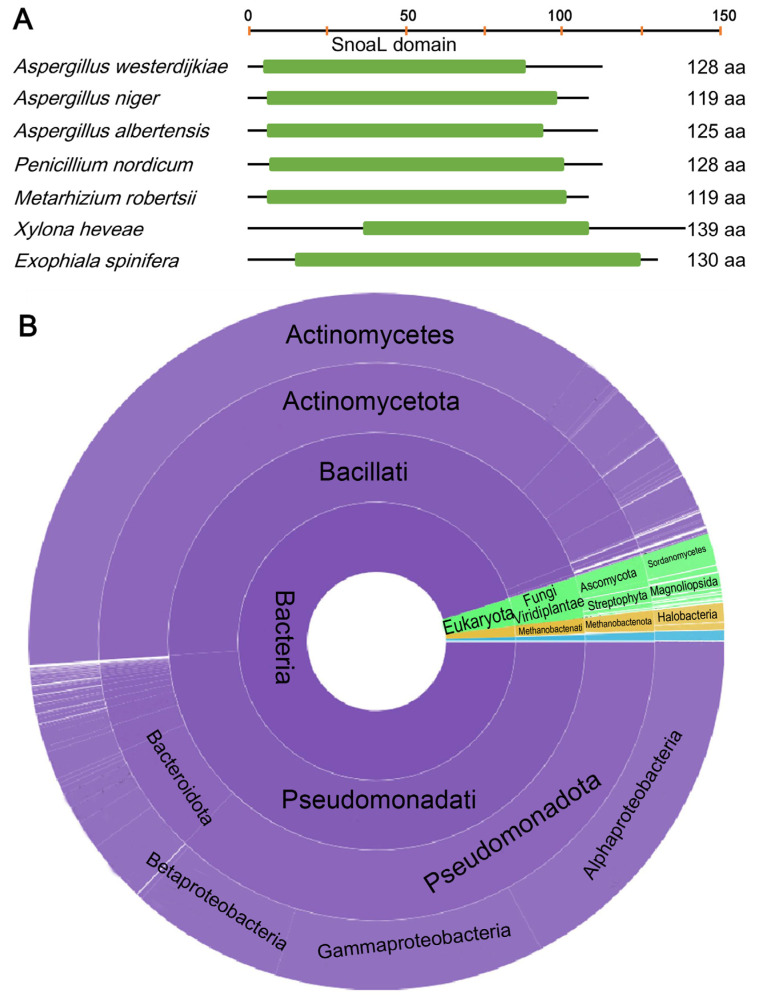
Homologs of OtaY in fungi and bacteria. (**A**) Domain analysis of OtaY homologs. (**B**) Distribution of SnoaL-containing proteins.

We hypothesize that OT evolved independently in *Aspergillus* and *Metarhizium*. It is difficult to determine the origin of this BGC, given the limitations of our understanding about microbial gene resources. Previous analyses improved the annotation of the OTA-producing species and led to the identification of *otaY* in this cluster [7]. The cyclase, containing a SnoaL domain, gives some insights into the original source of the OTA cluster, or of this gene in particular. The SnoaL family, comprising small polyketide cyclases with an average length of 140 amino acid residues, has been identified as mediating critical cyclization reactions during the biosynthesis pathway of polyketide SMs in *Streptomyces* [24,25]. The sequence length of OtaY ranged from 115 to 164 amino acids according to the ochratoxigenic fungi, and all the encoding genes contain no intron (Figure 7A). An EMBL-EBI database retrieval showed that 1.8% (1.9 K) of SnoaL-containing proteins (104 K) originated from fungi, while 94.7% of them were found to come from bacteria (Figure 7B). In bacteria, the majority of SnoaL proteins identified thus far originate from *Pseudomonadota*, *Actinomycetota* and *Bacteroidota*; in fungi, they are primarily found in *Sordariomycetes*, *Eurotiomycetes* and *Dothideomycetes* within the phylum *Ascomycota*.

**Figure 8:**
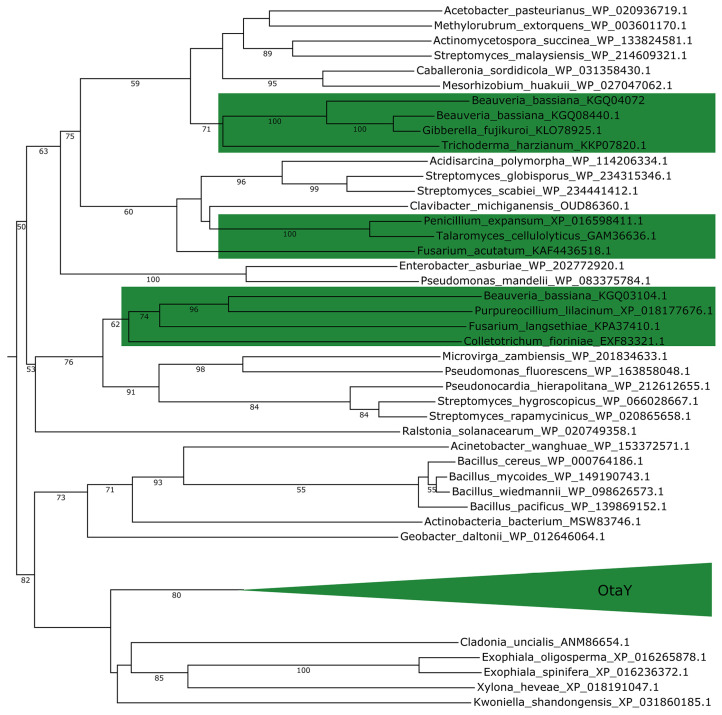
Phylogenetic analysis of SnoaL-containing proteins. Green background indicates proteins from fungal genomes.

A phylogenetic analysis was conducted using OtaY homologous proteins derived from fungal and bacterial sources. As illustrated in Figure 8, OtaY proteins from OTA-producing strains exhibited high sequence identity and clustered into a distinct clade. This clade demonstrated significant homology with SnoaL-like proteins of bacterial origin, including those from *Cladonia uncialis* and *Exophiala* spp. Homologs from fungi such as *Beauveria bassiana* and *P. expansum* were similarly clustered into branches alongside bacterial-derived proteins. These findings indicate that SnoaL-containing proteins are widely distributed in bacteria, and suggest HGT as the evolutionary mechanism through which fungi acquired *otaY* from bacterial lineages.

## 3. Discussion

The evolutionary trajectory of OTA biosynthesis remains an intriguing yet unresolved question in microbial genomics. In this study, phylogenetic reconstruction of OTA BGCs revealed unexpectedly high sequence conservation among phylogenetically divergent fungal species. Notably, key enzymes involved in OTA production exhibited >40% amino acid sequence identity, whereas flanking genomic regions beyond the OTA BGC showed minimal homology. This genomic dichotomy strongly suggests that HGT is a potential mechanism for OTA pathway dissemination in fungi. The frequent co-isolation of *Penicillium* and *Aspergillus* species from shared ecological niches, such as mold-contaminated foodstuffs and agricultural soils, provides plausible environmental contexts for such interspecies genetic exchanges.

HGT was first described in 1947 [26], when it was speculated to have a role in the ecological adaption of eukaryotes [27]. “First do no harm” has been an important role for transferred genes that are successfully integrated into a recipient [28]. It was also proposed that when the transferred genes remain neutral and provide no benefit associated with their retention, the genes would be lost over time [29]. The loss of OTA genes was evidenced by the occurrence of partial clusters, including species in *Aspergillus* section *Circumdati* and *Nigri*. Intraspecies variation was also observed in *Aspergillus niger*. Furthermore, the pattern of gene loss varied even in closely related species, and the break point of *otaA* genes also occurred at different loci. Collectively, these findings support the hypothesis that the OTA BGC is undergoing progressive decay in *Aspergillus* spp.

Understanding the ecological rationale for OTA biosynthesis in fungi remains critical for deciphering its evolutionary persistence and mitigating agricultural contamination. A few studies have suggested that OTA helps *Penicillium* species adapt to specific environments. For example, it was proposed that constant biosynthesis and excretion of the chlorinated OTA ensures a partial chloride homeostasis in *Penicillium nordicum* and *P. verrucosum*, which can be found as contaminants on NaCl-rich fermented foods, while most pathogenic or spoilage microbes are quite sensitive to high concentrations of NaCl [30,31]. OT BGC variation has not been detected in *Penicillium* spp., which could be associated with a smaller number of sequence strains or with the putative role of maintaining a chlorine balance under NaCl-rich environments. Although *P. chrysogenum* CGMCC 3.15265, *P. glycyrrhizacola* CGMCC 3.15273 and *P. polonicum* CGMCC 3.15264 and CGMCC 3.15272 have been reported to produce OTA [32], we could not detect any OT genes via the genome sequencing of these strains (Table S2). However, OTA is produced by several species regardless of Cl^−^ concentration in the environment. OTA could not help *Aspergillus westerdijkiae* adapt to chlorine stress, which was inferred from the fungal growth of wild-type otaA and the OT-disrupted mutant Δ*otaA* on varying salt regimes (Figure S3).

The characterization of *otaY* offers valuable insights into elucidating the origin of OTA in fungal species. SnoaL-containing proteins have been shown to catalyze an aldol condensation reaction for the ring closure steps in the biosynthesis of polyketides in *Streptomyces* [24,25], but the function of homologous proteins in fungi remains unclear. The deletion of *otaY* led to the absence of OTA in *Aspergillus carbonarius*, which points out the involvement of *otaY* in the biosynthetic pathway [8]. Another example of inclusion of this type of activity in fungal SMs comes from a study in which the deletion of the SnoaL-like enzyme AurE reduced yields of aurovertins in *Calcarisporium arbuscula*; in addition, AurE enhances the rate of pyrone formation in a yeast reconstitution system, indicating its predicted role as a cyclase [33]. Although these studies provide evidence of a functioning cyclase in fungi, the catalytic mechanisms for the cyclization reaction need to be further explored. Indeed, fungi employ a different cyclization mode compared with bacteria. Generally, nonreducing PKS catalase polyketides contain aromatic rings. In this case, the PT domain bridges an even-numbered carbanion carbon and an odd-numbered carbonyl carbon to complete the first ring closure, while the TE/CLC domain directs additional cyclization and offloads the backbone chain from the megasynthase [34,35]. As with other highly reducing PKSs, *otaA* is theoretically incapable of synthesizing aromatic polyketides due to the lack of PT and TE/CLC domains [36]. For this case, aromatic cyclization is thought to occur spontaneously [37]. Given the putative function of *otaY* and its necessity in OTA biosynthesis, we speculate that *otaY* is involved in the cyclization process via a bacterial catalytic mode. From this perspective, we speculate that *otaY* was horizontally transferred from bacteria to fungi.

Overall, the HGT of OT cluster homologs was evident from the phylogenetic analysis, and different patterns of gene loss in the OT cluster have occurred in *Aspergillus* species over time due to evolution. Furthermore, the rare presentation of *otaY* in fungi indicated its bacterial origin by HGT. For the first time, these results give insight into the ecological significance of OTA and suggest that this mycotoxin plays at best a neutral role in *Aspergillus* spp., with the possible exception of the salt-dwelling Penicillia.

## 4. Conclusions

Using phylogenetic analysis of OT genes, we found that *Aspergillus* and *Metarhizium* acquired an ancient OT cluster that developed independently, subsequently leading to the horizontal transfer of the OT cluster from *Aspergillus* to *Penicillium*. In addition, the cyclase *otaY* in the OTA cluster is likely derived from bacteria. The diverse occurrence of functional, absent and pseudogenized OT genes among *Aspergillus* species indicates that this cluster is experiencing a process of degeneration within this genus.

## 5. Materials and Methods

### 5.1. Strains and Culture Conditions

The fungal strains were grown at 28 °C on Potato Dextrose Agar (PDA, Becton, Dickinson and Company, Franklin Lakes, NJ, USA) plates. *Aspergillus westerdijkiae* Δ*otaA* was stored in the Institute of Food Science and Technology, Chinese Academy of Agricultural Sciences [6]. Mycelium tissues of the *Aspergillus* and *Penicillium* species, cultivated in the liquid Potato Dextrose Broth (PDB, Becton, Dickinson and Company), were harvested via filtration. Genomic DNA for sequencing was isolated using a Genomic DNA Kit (DP305, TIANGEN, Beijing, China).

### 5.2. Bioinformatic Analysis

The genomic data of strains were acquired from the NCBI and JGI databases. The raw sequencing data (Illumina HiSeq 2500) of nine strains in Table S1 were generated by Berry Genomics Co., Ltd. (Beijing, China). The genomes were assembled using SPAdes [38], and the genes were predicted using the Augustus algorithm [39]. The accession numbers of these new genomes were listed in Table S1. We used Benchmarking Universal Single-Copy Orthologs (BUSCO) v5.8.2 [39] to evaluate quality of assemblies used in this study. The results revealed that the BUSCO completeness of all the assemblies was above 92.20% (Table S1).

### 5.3. Phylogenetic Analysis

For the phylogenomic analysis of the fungal species used in this study, groups of proteins were detected by OrthoFinder v2.4.0 [40]. MUSCLE was used to align the single-copy orthologues [41], and then Gblocks was used to trim the poor alignment regions [42]. The high-quality sequences were employed for the maximum likelihood phylogeny analysis using RAxML-NG v. 0.5.1b [43]. Bootstrap support values were calculated by analyzing 1000 replicates. For the phylogeny analysis of OT proteins, the sequences were aligned using MUSCLE, and then a maximum likelihood tree was constructed using treeBeST (http://treesoft.sourceforge.net/treebest.shtml (accessed on 10 December 2024)) using 1000 bootstrap replicates [44]. The structural annotation of protein domains was performed utilizing the InterPro bioinformatics resource (https://www.ebi.ac.uk/interpro/ (accessed on 10 December 2024)).

### 5.4. Estimation of Gene Decay Rates

We determined the three gene states as functional (F), absent (A) and pseudogenized (P). The pseudogene included truncated and disrupted genes. The transition rates of gene states across *Aspergillus* species were calculated using BayesTraits V3.0.5 [45,46]. First, a species phylogenetic tree was constructed based on 2130 single-copy genes using RAxML-NG with 500 bootstrap replicates. The single-copy genes were detected using Orthofinder. Second, the 500 trees inferred from the RAxML-NG bootstrap replicates were used for running a BayesTraits analysis of each of the OT genes. The MultiState method and the maximum likelihood analysis were selected. Similar to the previous study [14], three models (H_0_, H_1_ and H_2_) of rate transitions were analyzed.

## Data Availability

The original contributions presented in this study are included in the article/Supplementary Material. Further inquiries can be directed to the corresponding authors.

## Supplementary Materials

The following supporting information can be downloaded at https://www.mdpi.com/article/10.3390/toxins17060311/s1: Figure S1: Phylogenomic relationships were inferred from 102 single-copy orthologs present in 71 genomes, and red asterisk indicated the bootstrap values were more than 50; Figure S2: Phylogenetic relationship of six OT genes; Figure S3: The colony view (A) and diameter (B) of *A. westerdijkiae* wild type and Δ*otaA* on YES media containing NaCl (g/L); Table S1: Fungal genome used in this study. The fungal strains sequenced in this study are indicated by asterisks; Table S2: Mean transition rates calculated by BayesTraits using three gene transition models.
