# Supplementary material for: Differential Retention and Loss of a Mycotoxin in Fungal Evolution
Source: Toxins (Basel). 2025 Jun 19;17(6):311. doi: 10.3390/toxins17060311 (PMC12197560; doi:10.3390/toxins17060311)
Supplement: Supplementary file 1 [file toxins-17-00311-s001.zip › toxins-3574729-supplementary.pdf]

Supplementary Materials: Differential Retention and Loss of a Mycotoxin in Fungal Evolution

Lin Chen, Ziying Yan, Bolei Yang, Bowen Tai, Weizhao Li, Erfeng Li, Gang Wang and Fuguo Xing

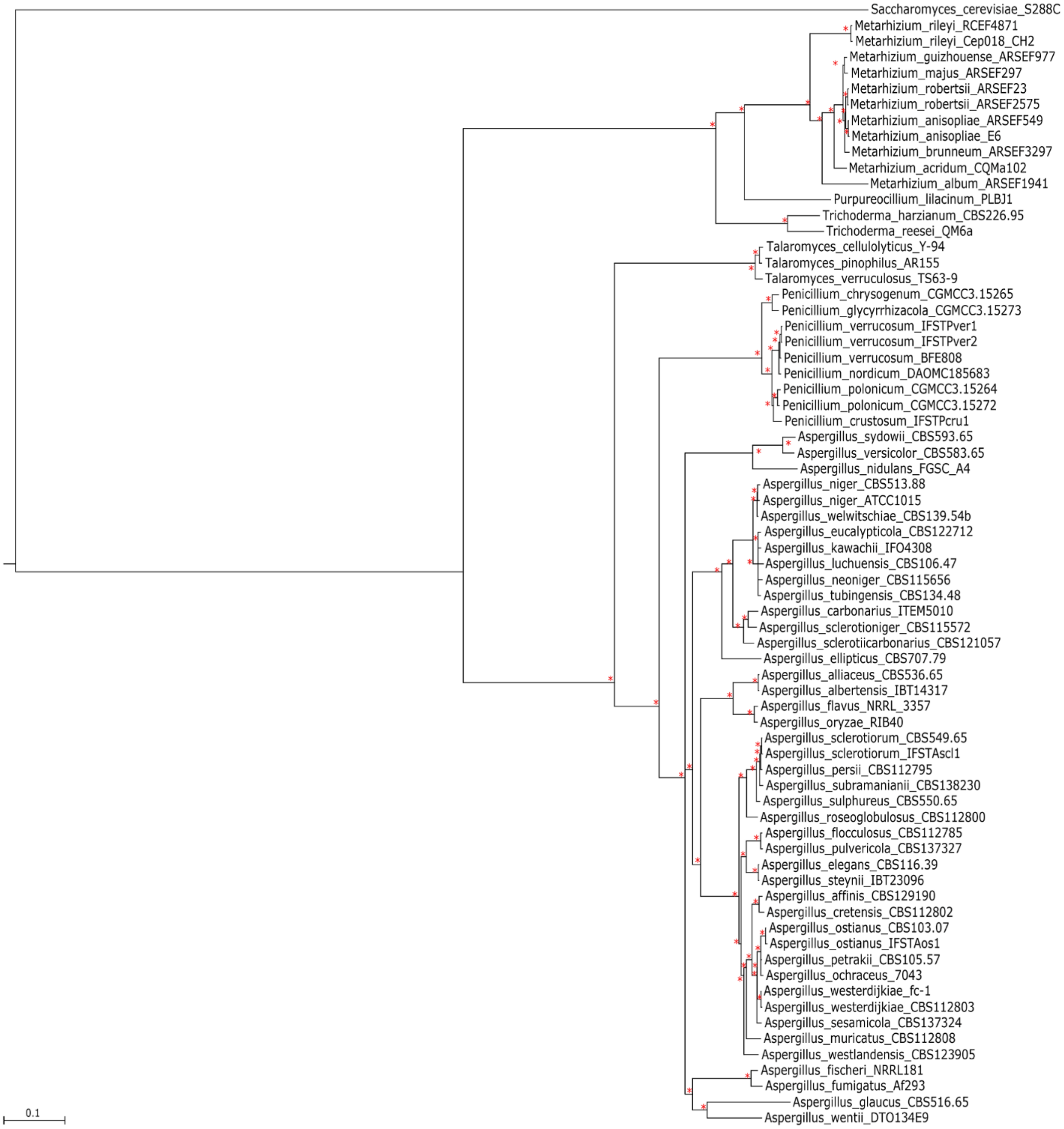

Figure S1. Phylogenomic relationships were inferred from 102 single-copy orthologs present in 71 genomes, and red asterisk indicated the bootstrap values were more than 50.

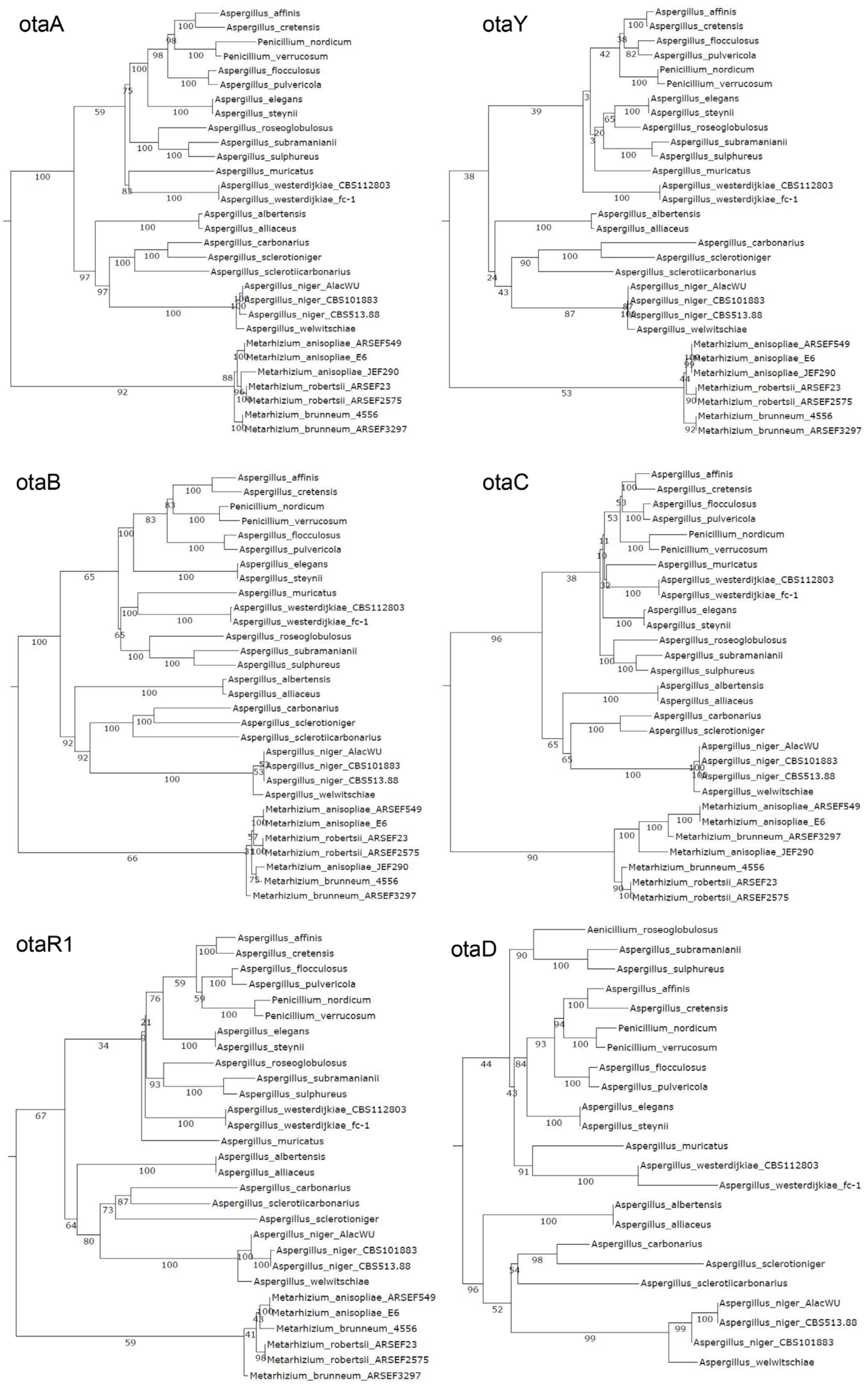

Figure S2. Phylogenetic relationship of six OT genes.

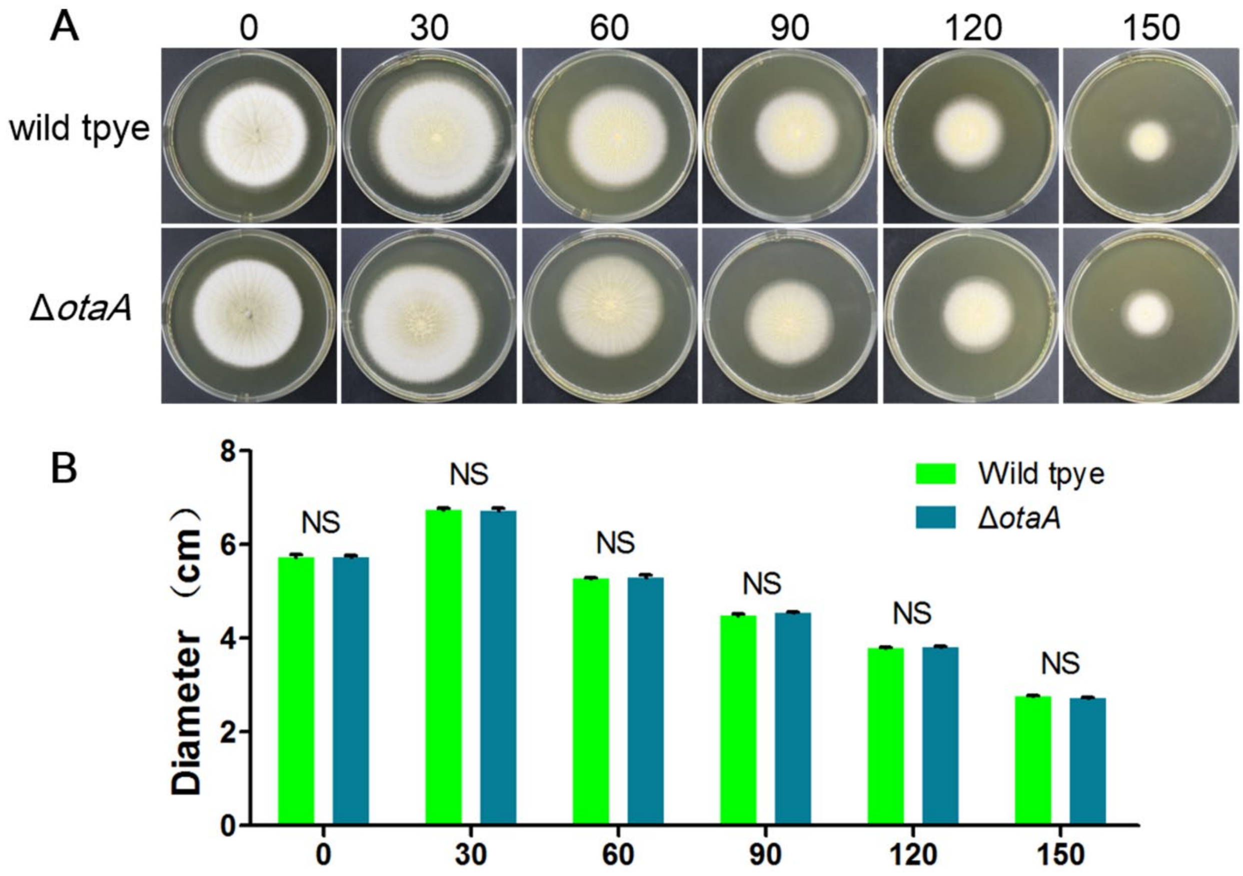

Figure S3. The colony view (**A**) and diameter (**B**) of A. westerdijkiae wild type and ΔotaA on YES media containing NaCl (g/L).

Table S1. Fungal genome used in this study. The fungal strains sequenced in this study are indicated by asterisks.

**Species**	**Strain**	**Dababase**	**Accession No.**	**Completeness**
**Aspergillus ostianus*[2]	IFST Aos1	NCBI	GCA_025592935.1	99.10%
**Aspergillus sclerotiorum*[1]	IFST Ascl1	NCBI	GCA_025592505.1	98.20%
**Penicillium chrysogenum*	CGMCC 3.15265	NCBI	GCA_025590035.1	99.20%
**Penicillium crustosum*	IFST Pcru1	NCBI	GCA_025583935.1	99.30%
**Penicillium glycyrrhizacola*	CGMCC 3.15273	NCBI	GCA_025586815.1	99.20%
**Penicillium polonicum*	CGMCC 3.15264	NCBI	GCA_025590805.1	99.60%
**Penicillium polonicum*	CGMCC 3.15272	NCBI	GCA_025589915.1	99.50%
**Penicillium verrucosum*	IFST Pver1	NCBI	GCA_025585045.1	99.60%
**Penicillium verrucosum*	IFST Pver2	NCBI	GCA_025585095.1	99.50%
Aspergillus affinis	CBS 129190	JGI	1052342	98.70%
Aspergillus albertensis	IBT 14317	JGI	1052228	98.90%
Aspergillus alliaceus	CBS 536.65	JGI	1051918	98.70%
Aspergillus carbonarius	ITEM 5010	JGI	1184803	95.00%
Aspergillus cretensis	CBS 112802	JGI	1052350	99.50%
Aspergillus elegans	CBS 116.39	JGI	1052356	98.40%
Aspergillus ellipticus	CBS 707.79	NCBI	GCA_003184645.1	98.70%
Aspergillus eucalypticola	CBS 122712	NCBI	GCF_003184535.1	98.90%
Aspergillus fischeri	NRRL181	NCBI	GCF_000149645.3	98.90%
Aspergillus flavus	NRRL_3357	NCBI	GCF_014117465.1	94.60%
Aspergillus flocculosus	CBS 112785	JGI	1052366	98.30%
Aspergillus fumigatus	Af293	NCBI	GCF_000002655.1	98.50%
Aspergillus glaucus	CBS 516.65	NCBI	GCF_001890805.1	99.70%
Aspergillus kawachii	IFO 4308	NCBI	GCF_016861625.1	98.50%
Aspergillus luchuensis	CBS 106.47	NCBI	GCA_001890685.1	95.60%
Aspergillus muricatus	CBS 112808	JGI	1052368	98.80%
Aspergillus neoniger	CBS 115656	NCBI	GCF_003184625.1	99.50%
Aspergillus nidulans	FGSC A4	NCBI	GCF_000011425.1	98.30%
Aspergillus niger	CBS 101883	JGI	1027297	99.20%
Aspergillus niger	AlacWU	NCBI		99.60%
Aspergillus niger[1]	CBS 513.88	NCBI	GCA_000002855.2	95.30%
Aspergillus niger[2]	ATCC1015	NCBI	GCA_000230395.2	96.80%
Aspergillus ochraceus	ITEM 7043	JGI	1266560	99.30%
Aspergillus oryzae	RIB40	NCBI	GCA_000184455.3	93.00%
Aspergillus ostianus[1]	CBS103.07	JGI	1052386	98.90%
Aspergillus persii	CBS 112795	JGI	1052392	99.30%
Aspergillus petrakii	CBS 105.57	JGI	1052398	99.20%
Aspergillus pulvericola	CBS 137327	JGI	1188009	98.80%
Aspergillus roseoglobulosus	CBS 112800	JGI	1052404	99.10%
Aspergillus sclerotiicarbonarius	CBS 121057	JGI	1027246	99.30%

Aspergillus sclerotioniger	CBS 115572	JGI	1027242	99.70%
Aspergillus sclerotiorum[2]	CBS 549.65	JGI	1051930	99.50%
Aspergillus sesamicola	CBS 137324	JGI	1188013	98.30%
Aspergillus steynii	IBT 23096	NCBI	GCA_002849105.1	99.50%
Aspergillus subramanianii	CBS 138230	JGI	1188017	99.50%
Aspergillus sulphureus	CBS 550.65	JGI	1052416	99.30%
Aspergillus sydowii	CBS593.65	NCBI	GCA_001890705.1	99.60%
Aspergillus tubingensis	CBS 134.48	NCBI	GCA_001890745.1	99.20%
Aspergillus versicolor	CBS583.65	NCBI	GCA_001890125.1	99.30%
Aspergillus welwitschiae	CBS 139.54b	JGI	1060057	98.40%
Aspergillus wentii	DTO 134E9	NCBI	GCA_001890725.1	99.20%
Aspergillus westerdijkiae[1]	fc-1	NCBI	GCA_004849945.1	98.90%
Aspergillus westerdijkiae[2]	CBS 112803	JGI	1188021	98.70%
Aspergillus westlandensis	CBS 123905	JGI	1097346	98.70%
Metarhizium acridum	CQMa102	NCBI	GCA_000187405.1	95.80%
Metarhizium album	ARSEF1941	NCBI	GCF_000804445.1	97.60%
Metarhizium anisopliae	JEF-290	NCBI		92.20%
Metarhizium anisopliae	ARSEF 549	NCBI	GCA_000814975.1	97.40%
Metarhizium anisopliae	E6	NCBI	GCA_000739145.1	98.20%
Metarhizium brunneum	ARSEF3297	NCBI	GCA_000814965.1	97.10%
Metarhizium brunneum	4556	NCBI	GCA_013426205.1	99.50%
Metarhizium guizhouense	ARSEF977	NCBI	GCA_000814955.1	97.10%
Metarhizium majus	ARSEF297	NCBI	GCA_000814945.1	96.80%
Metarhizium rileyi	RCEF4871	NCBI	GCA_001636745.1	98.40%
Metarhizium rileyi	Cep018 CH2	NCBI	GCA_007866325.1	98.90%
Metarhizium robertsii	ARSEF2575	NCBI	GCA_000591435.1	98.70%
Metarhizium robertsii	ARSEF 23	NCBI	GCA_000187425.2	99.20%
Penicillium nordicum	DAOMC 185683	NCBI	GCA_001278595.1	98.20%
Penicillium verrucosum	BFE808	NCBI	GCA_000970515.2	98.90%
Purpureocillium lilacinum	PLBJ1	NCBI	GCA_001653265.1	96.70%
Saccharomyces cerevisiae	S288C	NCBI	GCA_000146045.2	96.70%
Talaromyces cellulolyticus	Y-94	NCBI	GCA_000829775.1	97.20%
Talaromyces pinophilus	AR155	NCBI	GCA_011392495.1	96.40%
Talaromyces verruculosus	TS63-9	NCBI	GCA_001305275.1	97.40%
Trichoderma harzianum	CBS226.95	NCBI	GCA_003025095.1	99.90%
Trichoderma reesei	QM6a	NCBI	GCA_000167675.2	99.60%

Table S2. Mean transition rates calculated by BayesTraits using three gene transition models.

	Tree No	Lh	q(A to F)	q(A to P)	q(F to A)	q (F to P)	q(P to A)	q (P to F)
H_0	otaA	-25.46	0.14	0.00	6.40	35.20	0.10	99.98
	otaY	-22.95	60.58	--	60.10	--	--	--
	otaB	-25.91	63.00	0.06	55.10	2.70	16.47	4.35
	otaC	-27.37	33.36	0.00	30.71	14.64	0.00	99.76
	otaR1	-19.96	16.35	--	14.24	--	--	--
	otaD	-22.11	6.84	0.00	11.35	3.78	0.32	72.20
H_1	otaA	-31.28	26.88	0.00	10.31	14.65	29.14	0.00
	otaY	-22.95	60.58	--	60.10	--	--	--
	otaB	-25.91	63.37	0.00	55.24	2.59	17.75	0.00
	otaC	-28.87	54.24	0.00	37.29	10.62	65.76	0.00
	otaR1	-19.96	16.35	--	14.24	--	--	--
	otaD	-24.44	22.77	0.00	17.27	2.30	32.48	0.00
H_2	otaA	-31.52	0.00	0.00	0.13	12.12	31.22	0.00
	otaY	-27.98	0.00	--	12.55	--	--	--
	otaB	-31.24	0.00	0.00	11.25	1.29	0.76	0.00
	otaC	-34.30	0.00	0.00	8.23	3.81	33.92	0.00
	otaR1	-22.29	0.00	--	7.86	--	--	--
	otaD	-26.99	0.00	0.00	6.85	2.28	21.72	0.00
